# Hemolytic Uremic Syndrome Due to Methylmalonic Acidemia and Homocystinuria in an Infant: A Case Report and Literature Review

**DOI:** 10.3390/children8020112

**Published:** 2021-02-05

**Authors:** Vasiliki Karava, Antonia Kondou, John Dotis, Georgia Sotiriou, Spyridon Gerou, Helen Michelakakis, Euthymia Vargiami, Marina Economou, Dimitrios Zafeiriou, Nikoleta Printza

**Affiliations:** 1 1st Department of Pediatrics, Aristotle University of Thessaloniki, Hippokratio General Hospital, 546 42 Thessaloniki, Greece; vasilikikarava@hotmail.fr (V.K.); tkondou@hotmail.com (A.K.); yan_dot@yahoo.com (J.D.); gmsoti@gmail.com (G.S.); evargiam@auth.gr (E.V.); kely@auth.gr (M.E.); zafeirioudi@gmail.com (D.Z.); 2 Gerou Analysis Medical S.A., Diagnostic-Research Clinics, 546 22 Thessaloniki, Greece; analysi@analysi.gr; 3 Department of Enzymology and Cellular Function, Institute of Child Health, 115 26 Athens, Greece; ecfdept@ich.gr

**Keywords:** hemolytic uremic syndrome, thrombotic microangiopathy, cobalamic C defect, methylmalonic acidemia and homocystinuria, early-onset cblC deficit

## Abstract

Methylmalonic acidemia and homocystinuria cobalamin C (cblC) type is the most common inborn error of the intracellular cobalamin metabolism, associated with multisystem involvement and high mortality rates, especially in the early-onset form of the disease. Hemolytic uremic syndrome (HUS) is a rare manifestation and needs to be distinguished from other causes of renal thrombotic microangiopathy. We describe a case of a 3-month-old infant, with failure to thrive, hypotonia and pallor, who developed HUS in the setting of cblC deficit, along with dilated cardiomyopathy, and presented delayed response to optic stimulation in visual evoked potentials, as well as enlarged bilateral subarachnoid spaces and delayed myelination in brain magnetic resonance imaging. Renal damage was reversed, while neurodevelopmental profile and eye contact improved after supplementation with parenteral hydroxycobalamin, oral folic acid, betaine and levocarnitine. Homozygous mutation of c.271dupA in the MMACHC gene was ultimately detected. In this report, we highlight the diagnostic challenges as well as the significance of early recognition and multidisciplinary management of this unusual condition. A brief review of published case reports of early-onset cblC deficit and related HUS is depicted, pointing out the initial clinical presentation, signs of renal damage and outcome, MMACHC gene type of mutations and accompanying extra-renal manifestations.

## 1. Introduction

Combined methylmalonic acidemia and homocystinuria is an inherited disorder of the intracellular cobalamin metabolism, due to mutations in several genes, including MMACHC, MMADHC, LMBRD1, ABCD4, or HCFC1; each gene mutation corresponds to a different type of the disease [1]. Cobalamin C (cblC) complementation type of the disease is caused by mutations in the MMACHC gene, located on chromosome 1p34.1. The disease is inherited in an autosomal recessive manner and is considered the most frequent inborn error of intracellular cobalamin metabolism, with an incidence varying from 1/37,000 to 1/200,000 births among different populations [2]. Sequence analysis of MMACHC is currently suggested as the first step in the genetic diagnosis of patients with biochemical findings of combined methylmalonic acidemia and homocystinuria, while multigene panel analysis is reserved for cases with non-diagnostic single-gene testing [1]. Pathogenetic mechanism of this disorder consists of deficient decyanation of cyanocobalamin and downstream cytoplasmic blocking of cobalamin conversion into its two metabolically active forms, adenosylcobalamin and methylcobalamin. Adenosylcobalamin and methylcobalamin are essential cofactors of the enzymes methylmalonyl-coenzyme A (CoA) mutase and methionine synthetase, located in cytoplasm and mitochondria, respectively. Compromised function of these enzymes due to cblC deficiency leads to reduced degradation of methylmalonyl-CoA to succinyl-CoA and reduced remethylation of homocysteine to methionine. As a result, increased levels of homocysteine in plasma and of MMA in plasma and urine are highly indicative biochemical markers of the disease [3]. 

The majority of cblC defect-related clinical symptoms occur during the neonatal period or infancy (<12 months of age), corresponding to the early-onset form of the disease and accounting for approximately 90% of reported cases [4]. Nevertheless, signs of this metabolic disorder may be present even in utero, while late-onset case-series during childhood or adulthood have also been reported [2]. Disorder of intracellular cobalamin metabolism due to cblC defects is characterized by a large-spectrum phenotype, reflecting the impaired MMACHC expression and accumulated homocysteine and MMA in several tissues, which varies by age of disease onset. Neuropsychiatric symptoms, progressive cognitive decline and thromboembolic complications constitute the most common clinical presentation in the late-onset forms of the disease, while multisystem disorders, comprising of growth retardation, dysmorphic facial features and manifestations from various organs, including central nervous system, eye, blood, vessels, heart, kidney, liver, gastrointestinal tract and skin, are primarily present in the early-onset disease [3]. Unfavorable outcome and mortality are most often observed in the early-onset type of the disease [5]. Early and adequate treatment with parenteral hydroxycobalamin has been proven to be beneficial for the resolution of hematological abnormalities and improvement in the systemic disturbances of the disease [2]. Oral supplementation with betaine is also recommended for reduction of plasma homocysteine levels, while additional therapy with levocarnitine and folic acid are usually prescribed for enhancement of carnitine and methionine synthesis, which may be impaired in these patients [2].

Renal manifestations of the disease have rarely been observed, in either early or late-onset forms, and mainly include renal thrombotic microangiopathy (TMA), eventually leading to hemolytic uremic syndrome (HUS) [6]. Tubulointerstitial nephritis [7] and proximal renal tubular acidosis have occasionally been reported [8]. Fibrin thrombi in glomerular capillaries, endothelial swelling, thickening and detachment of glomerular basement membrane are common renal pathology findings [6]. The pathogenetic mechanism of renal TMA in the setting of cblC defect is not completely understood. Experimental studies have shown that homocysteine may directly cause endothelial injury and stimulate platelet prostaglandin synthetase, favoring vascular thrombosis [9,10]. Nevertheless, the absence of renal injury in other inborn errors of intracellular cobalamin disorders with high homocysteine levels indicate that other factors are probably required to induce renal TMA in cblC disorders.

In the present report, we describe an unusual case of infantile renal HUS secondary to cblC defects, emphasizing various diagnostic challenges. Moreover, we discuss the current literature regarding the initial clinical symptoms, signs of renal damage and outcome, the MMACHC type of genetic mutations and accompanied extra-renal manifestations of patients with early-onset disease and associated renal damage.

## 2. Case Report

The index patient was a full-term male, born by a normal spontaneous vaginal delivery to a G1P1 mother, with good clinical status at birth; Apgar scores were 8 and 9 at 1 and 5 min, respectively. His birth weight, height and head circumference were 2880 gr (15th perc), 48 cm (15th perc) and 35 cm (50th perc), respectively. The pregnancy was uncomplicated, while the prenatal growth and organ development were normal, according to fetal ultrasounds. No parental consanguinity was reported, and family history was negative for genetically determined disorders. 

According to the mother’s report, at two months of age, the infant presented with failure to thrive, corresponding to a 10gr month weight gain, and was more somnolent and paler than usual. One month later, the infant underwent serum and urine laboratory tests, which revealed severe normocytic normochromic anemia (hemoglobin: 6 gr/dl, hematocrit: 16%, mean corpuscular volume: 95.7 fL, mean corpuscular hemoglobin: 33.7 pg), of hemolytic non-immune origin (negative direct Coombs test, schistocytes: 4%, reticulocyte count: 9%, lactate dehydrogenase: 868 UI/L, total bilirubin: 2.21 mg/dL, direct bilirubin:0.32 mg/dL) combined with thrombocytopenia (platelets:141,000/μL), nephrotic range proteinuria (urine protein to creatinine ratio: 4.7 g/g, albumin to creatinine ratio: 4100 mg/g), microscopic hematuria (blood cells: 30–35 per high power field) and normal kidney function (serum urea: 22 mg/dL, serum creatinine: 0.29 mg/dL). Diagnosis of renal TMA was established and the patient was admitted to hospital for further investigation. At initial examination, the patient was pale and presented with high blood pressure (BP) (BP: 134/83 mmHg, >99th perc), generalized central hypotonia of limbs, trunk and neck associated with increased peripheral muscle tone and absence of eye contact. 

Among the initial diagnostic tests, normal ADAMTS13 activity (ADAMTS13 activity: 96%) was indicative of absence of congenital thrombotic thrombocytopenic purpura (TTP). Typical hemolytic uremic syndrome (HUS) was excluded based on an absence of diarrhea history, negative polymerase chain reaction (PCR) for Shiga-toxins 1 and 2 and no growth of *Escherichia coli* O157:H7 or other pathogen in fecal specimen cultures. Normal serum C3 (serum C3: 0.75 g/L, reference range: 0.4–0.92 g/L), C4 (serum C4: 0.17 g/L, reference range: 0.1–0.37 g/L), and C5b-9 levels (serum C5b-9: 214 ng/mL, reference value <250 ng/mL) disassociated possible diagnosis from an alternative complement pathway dysregulation. In parallel, the high serum homocysteine level (serum homocysteine: 148 μmol/L, reference value <14 μmol/L) together with absence of cobalamin (cbl) (serum hydroxycobalamin level: 1119 pg/mL, deficient<211 pg/mL) and folate (serum folate 23.9 ng/mL, deficient <14 ng/mL) deficiency were suggestive of an inborn error of intracellular cobalamin metabolism. Treatment with hydroxycobalamin (1 mg/day, i.m.), betaine (1 g/day, p.o.), folic acid (5 mg/day, p.o.) and L-carnitine (1g/day p.o.) was initiated and screening of urinary organic acids was performed. High urinary methylmalonic acid (MMA) levels (urinary MMA/creatinine: 3482mmol/mmol, reference value <2.5 mmol/mmol) were indicative of combined methylmalonic acidemia and homocystinuria, cblC type. Of note, serum methionine levels were within the normal range (serum methionine: 28 μmol/L, reference value <40 μmol/L). The diagnosis was finally confirmed with whole exome sequencing analysis, which revealed homozygous mutation of c.271dupA (P.Arg91Lysfs*14) in the MMACHC gene. Both parents were heterozygous for the same mutation. 

**Figure 1:**
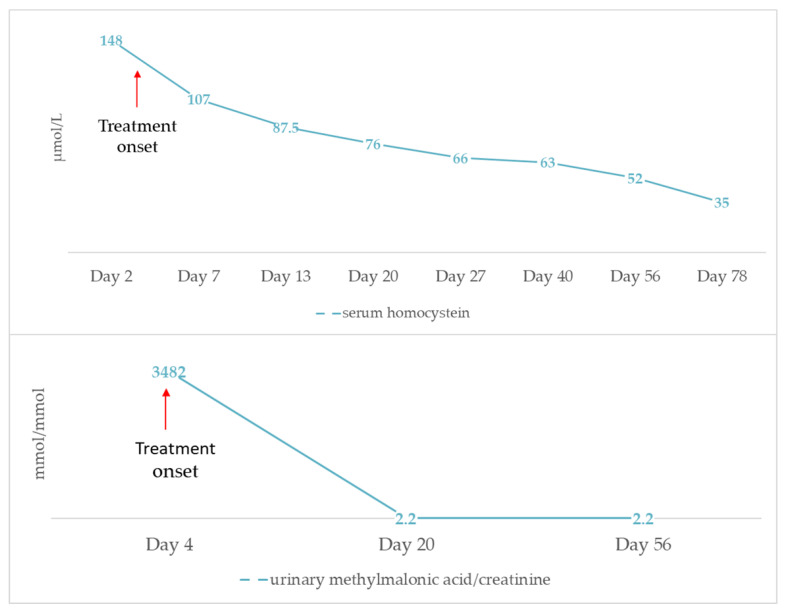
Progression of serum homocysteine and urinary methylmalonic acid levels after initiation of treatment with hydroxycobalamin, betain, folic acid and L-carnitine.

During the first week of hospitalization, serum urea and creatinine were slightly increased (serum urea max: 87 mg/dL, serum creatinine max: 0.71 mg/dL), indicating acute kidney injury (AKI). Both values returned to normal one week following treatment onset. Of note, diuresis was conserved with stable urine output around 3.5 mL/kg/h, while ultrasound imaging revealed normal size, hyperechogenic kidneys with normal corticomedullary differentiation. The patient also developed left ventricular dilated cardiomyopathy without pulmonary hypertension and with good myocardial contractility. Specifically, heart ultrasound revealed left ventricular internal dimension in diastole of 3 cm and left ventricular ejection fraction of 70%. Carvedilol (0.3mg × 2/day p.o) and furosemide were added to his treatment. Brain magnetic resonance imaging showed large bilateral subarachnoid spaces with ventricular system of normal size and delayed white matter myelination. Visual evoked potential testing demonstrated delayed response to optical stimulation. Of note, no signs of maculopathy were evidenced at ophthalmologic evaluation. Finally, we observed an abrupt fall of urinary MMA, which decreased to normal values approximately two weeks after treatment onset, whereas serum homocysteine levels slowly reduced, as illustrated in Figure 1. Vitamin B12 levels remained above 2000 pg/mL.

During the 9-month follow-up period, the patient preserved normal renal function. Proteinuria was resolved, while intermittent microscopic hematuria remained. Dilated cardiomyopathy regressed and relevant medication was, ultimately, discontinued. Both neurodevelopmental profile and eye contact gradually improved. 

## 3. Discussion

HUS is defined as the laboratory triad of non-autoimmune hemolytic anemia, thrombocytopenia and AKI, indicating the presence of renal TMA. Differential diagnosis in the neonatal period and infancy principally involves TTP due to congenital ADAMTS13 deficiency, atypical HUS related to alternative complement pathway dysregulation and HUS secondary to *Streptococcus pneumonia* or *Influenza A/H1N1* infection [11]. Less frequent causes are typical HUS triggered by *Shiga toxin-producing Escherichia coli (STEC)* infection, cblC disorder and diacylglycerol kinase ε (DKGE) gene mutation [11]. Taking into account the severity of this condition and the variable therapeutic management depending on the causative pathogenetic mechanism, a comprehensive diagnostic workup is necessary for prompt diagnosis of the underlying disease, as performed in the index patient. Therefore, in case of neonatal/infantile HUS, plasma homocysteine and plasma/urine methylmalonic acid level measurements are highly suggested in order to eliminate cblC defect. Nevertheless, symptoms of cblC defect may initially occur at an older age, while potential misdiagnosis as atypical HUS may result in unnecessarily prolonged treatment with complement blockage factor, delayed diagnosis and subsequent severe complications, such as pulmonary hypertension and relapse of AKI [12,13]. Interestingly, cblC defect has also been identified in a few patients with alternative complement pathway disorders, including an infant with low complement factor H (CFH) activity [14], a child with CFH mutation [15], a child with encoding membrane cofactor protein (CD46) mutation [16] and a young adult with CFH antibody-mediated HUS [17]. Therefore, it seems prudent to perform the widely available and low-cost homocysteine assay as part of the HUS diagnostic workup, regardless of age-onset, and even in cases with defined genetic or acquired alternative complement pathway dysregulation.

**Figure 2:**
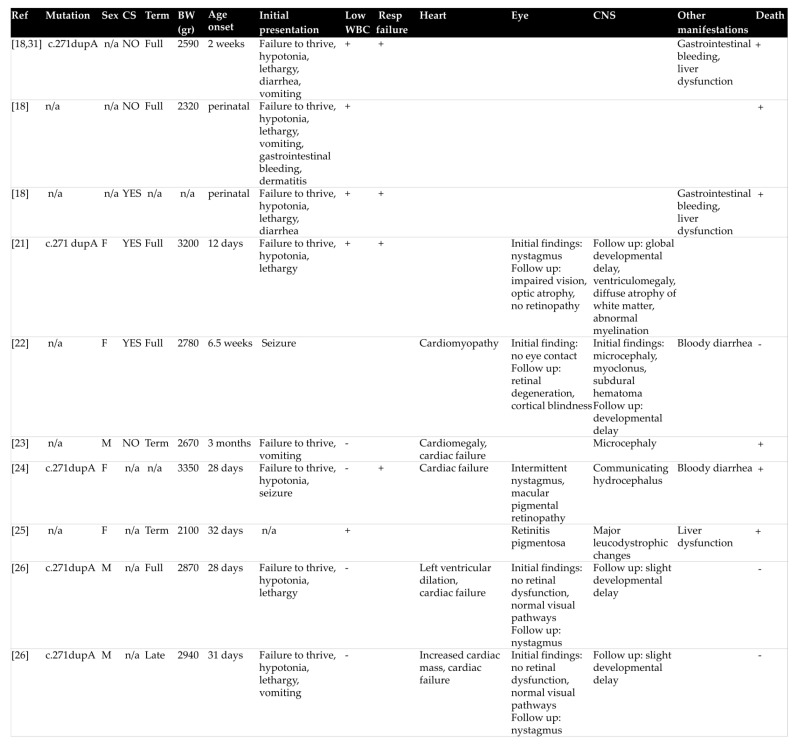

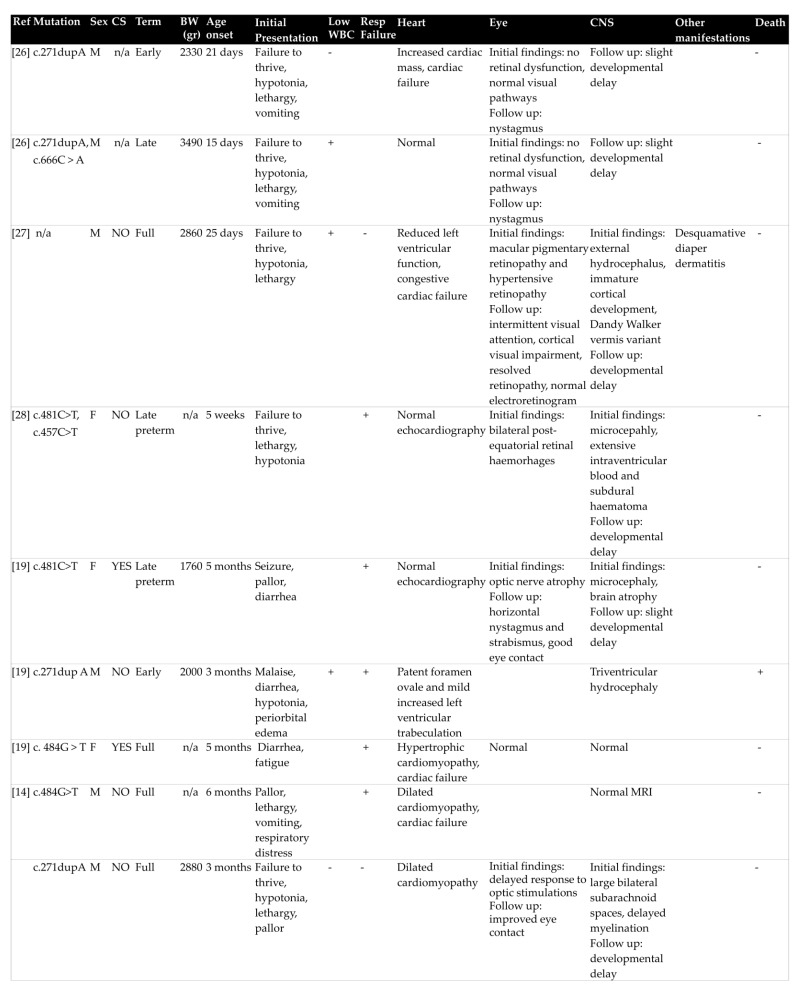
Dermographic parameters, perinatal history, initial presentation, extra-renal manifestations, type of MMACHC mutation and outcome of patients with early-onset cyanocobalamin C defect and hemolytic uremic syndrome. BW: birth weight, CNS: central nervous system, CS: consanguinity, WBC: white blood cells, MRI: magnetic resonance imaging, Resp failure: respiratory failure.

Renal damage in the setting of cblC disorder remains a rare condition in the neonatal period and infancy and has only occasionally been described in the literature. We therefore reviewed clinical and laboratory findings of 19 reported cases of early-onset cblC disorder and related renal TMA (Figure 2), more than half of which (52.6%) occurred during post-neonatal period [14,18,19,20,21,22,23,24,25,26,27,28].

**Figure 3:**
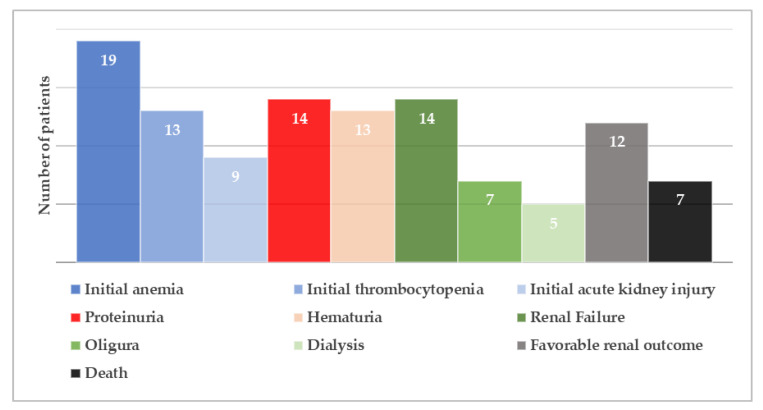
Renal manifestations at initial presentation and during the course of the disease of reported 19 patients with early-onset cobalamin C defect and hemolytic uremic syndrome.

Most patients were born at term and had a low to normal birth weight (median value: 2780 gr, range: 1760−3490 gr). Interestingly, parental consanguinity was not commonly reported (five cases in total). The main initial clinical presentations involved failure to thrive, hypotonia and lethargy, often associated with gastrointestinal symptoms and, occasionally, with seizures. It is worth mentioning that all three criteria of HUS were fulfilled on admission in only seven (36.8%) patients (Figure 3). In detail, while non-autoimmune hemolytic anemia was present in all cases, thrombocytopenia was observed in 13 (68.4%) and AKI in 9 (47.4%) patients, respectively. This observation raises attention for considering cblC defect-related renal TMA in all cases of microangiopathic hemolytic anemia, regardless of thrombocytopenia absence or serum creatinine rise. Moreover, careful search for proteinuria and hematuria is recommended in such cases, for early diagnosis of glomerular damage in the setting of possible renal TMA, as in the example of the patient previously described. In the case-series analysis, 14 (73.7%) patients progressed to renal failure, while oliguria and dialysis were reported in 7 (36.8%) and 5 (26.3%) cases, respectively. Setting aside cases with fatal outcome (7 patients, 36.8%), the renal outcome was favorable in all reported cases, suggesting that early administration of targeted treatment may reverse renal damage.

Molecular diagnosis of the disease is necessary in clinical practice both for confirmation of cblC type, as well as genetic counseling for future pregnancies. The MMACHC gene sequence in large patient series has shed light on possible phenotype–genotype correlations of the disease [29]. In general, frameshift mutation c.271dupA is present in approximately 40% of reported cblC defect cases in the Caucasian population, mostly observed in early-onset cases, succeeded by the nonsense mutation c.331C > T [29,30]. Homozygosity or compound heterozygosity for these two mutations constitutes the main genotype of early-onset disease [31]. Likewise, homozygous c.271dupA mutation was observed in most infants with HUS-related cblC disorder in this case series analysis. It is noteworthy that no case of c.331C > T mutation was recorded. In general, a correlation between genotype and age-onset is evidenced, with nonsense mutation c.394C > T representing the main mutation in mild late-onset disease [29]. Moreover, specific mutations are more common in certain ethnicities. For instance, nonsense mutation c.666C > A is commonly observed in patients with Italian origin, as was the origin of the one patient with the same identified mutation in the present review [29]. Furthermore, nonsense mutation c.481C > T was reported in one Turkish patient and one patient of Oriental origin [19,28,31] and homozygous missense mutation c. 484G > T in two other Turkish patients [14,20]. Interestingly, the latter mutation was not included in the common pathogenic variants of the disease. The role of these mutations in the various disease manifestations among different ethnicities, as well as the predisposition of c. 484G > T mutation for renal TMA, needs to be further investigated. 

Long-term multidisciplinary management of cblC disorder is important, especially in cases of early-onset of the disease, because of potential multisystem involvement and variable response to hydroxycobalamin treatment [2]. Most commonly affected organs, as highlighted in our review, involve the central nervous system, the heart and eye. Hypotonia, lethargy, microcephaly and seizures were the most common neurological manifestations, while central nervous system imaging findings included hydrocephalus, white matter involvement ranging from myelination delay to leukodystrophy and brain atrophy. Most frequently reported cardiac manifestations were dilated and hypertrophic cardiomyopathy, which may subsequently lead to heart failure. Nystagmus, strabismus and impaired eye contact were the main ocular clinical signs of the disease reported, while pigmented macular changes, macular degeneration, retinal dysfunction, delayed and reduced scotopic and photopic responses in visual evoked potentials, and, less frequently, optic nerve atrophy were encountered in some patients. Other disease manifestations included leucopenia (9 patients), gastrointestinal bleeding (5 patients), liver dysfunction (3 patients), dermatitis (2 patients) and respiratory failure (9 patients) necessitating mechanical ventilation.

## 4. Conclusions

Combined methylmalonic acidemia and homocystinuria is a potentially severe condition, which should be considered in cases of neonatal or infantile HUS. Renal damage may be present despite initial normal kidney function. Early diagnosis and treatment with parenteral hydroxycobalamin as well as a multidisciplinary approach are crucial for the optimal outcome of this rare disorder.

## References

[1] Sloan J.L., Carrillo N., Adams D., Venditti C.P., Adam M.P., Ardinger H.H., Pagon R.A., Wallace S.E., Bean L.J.H., Stephens K., Amemiya A. (2008). Disorders of Intracellular Cobalamin Metabolism. GeneReviews^®^.

[2] Carrillo-Carrasco N., Venditti C.P. (2012). Combined methylmalonic acidemia and homocystinuria, cblC type. II. Complications, pathophysiology, and outcomes. J. Inherit. Metab. Dis..

[3] Carrillo-Carrasco N., Chandler R.J., Venditti C.P. (2012). Combined methylmalonic acidemia and homocystinuria, cblC type. I. Clinical presentations, diagnosis and management. J. Inherit. Metab. Dis..

[4] Fischer S., Huemer M., Baumgartner M., Deodato F., Ballhausen D., Boneh A., Burlina A.B., Cerone R., Garcia P., Gökçay G. (2014). Clinical presentation and outcome in a series of 88 patients with the cblC defect. J. Inherit. Metab. Dis..

[5] Rosenblatt D.S., Aspler A.L., Shevell M.I., Pletcher B.A., Fenton W.A., Seashore M.R. (1997). Clinical heterogeneity and progno-sis in combined methylmalonic aciduria and homocystinuria (cblC). J. Inherit. Metab. Dis..

[6] Beck B.B., Van Spronsen F., Diepstra A., Berger R.M.F., Kömhoff M. (2017). Renal thrombotic microangiopathy in patients with cblC defect: Review of an under-recognized entity. Pediatr. Nephrol..

[7] Rutledge S.L., Geraghty M., Mroczek E., Rosenblatt D., Kohout E. (1993). Tubulointerstitial nephritis in methylmalonic acidem-ia. Pediatr. Nephrol..

[8] Wolff J.A., Strom C., Griswold W., Sweetman F., Kulovich S., Prodanos C., Nyhan W.L. (1985). Proximal Renal Tubular Acidosis in Methylmalonic Acidemia. J. Neurogenet..

[9] Graeber J.E., Slott J.H., Ulane R.E., Schulman J.D., Stuart M.J. (1982). Effect of Homocysteine and Homocystine on Platelet and Vascular Arachidonic Acid Metabolism. Pediatr. Res..

[10] Austin R.C., Lentz S.R., Werstuck G.H. (2004). Role of hyperhomocysteinemia in endothelial dysfunction and atherothrombotic disease. Cell Death Differ..

[11] Loirat C., Fakhouri F., Ariceta G., Besbas N., Bitzan M., Bjerre A., Coppo R., Emma F., Johnson S., Karpman D. (2016). An international consensus approach to the man-agement of atypical hemolytic uremic syndrome in children. Pediatr. Nephrol..

[12] Martínez de Compañón Z., Poblet-Puig M., Vallès G., Del Toro M., Vilalta R., Moreno A., Balcells J. (2018). Cobalamin disorder CblC presenting with hemolytic uremic syndrome and pulmonary hypertension. Nefrología.

[13] Gall E.C.-L., Delmas Y., De Parscau L., Doucet L., Ogier H., Benoist J.-F., Fremeaux-Bacchi V., Le Meur Y. (2014). Adult-Onset Eculizumab-Resistant Hemolytic Uremic Syndrome Associated with Cobalamin C Deficiency. Am. J. Kidney Dis..

[14] Barlas Ü.K., Kıhtır H.S., Göknar N., Ersoy M., Akçay N., Sevketoglu E. (2018). Hemolytic uremic syndrome with dual caution in an infant: Cobalamin C defect and complement dysregulation successfully treated with eculizumab. Pediatr. Nephrol..

[15] Guigonis V., Frémeaux-Bacchi V., Giraudier S., Favier R., Borderie D., Massy Z., Béatrice M., Rosenblatt D.S., Georges D. (2005). Late-onset thrombocytic microan-giopathy caused by cblC disease: Association with a factor H mutation. Am. J. Kidney Dis..

[16] Bouts A.H., Roofthooft M.T.R., Salomons G.S., Davin J.C. (2010). CD46-associated atypical hemolytic uremic syndrome with un-common course caused by cblC deficiency. Pediatr. Nephrol..

[17] Philipponnet C., Desenclos J., Brailova M., Aniort J., Kemeny J.-L., Talbot C., Frémeaux-Bacchi V., Souweine B., Heng A.-E. (2020). Cobalamin c deficiency associated with antifactor h antibody-associated hemolytic uremic syndrome in a young adult. BMC Nephrol..

[18] Russo P., Doyon J., Sonsino E., Ogier H., Saudubray J.-M. (1992). A congenital anomaly of vitamin B12 metabolism: A study of three cases. Hum. Pathol..

[19] Topaloglu R., Inözü M., Gülhan B., Gürbüz B., Talim B., Coşkun T. (2019). Do not Miss Rare and Treatable Cause of Early-Onset Hemolytic Uremic Syndrome: Cobalamin C Deficiency. Nephron.

[20] Adrovic A., Canpolat N., Caliskan S., Sever L., Kıykım E., Agbas A., Baumgartner M.R. (2016). Cobalamin C defect-hemolytic uremic syn-drome caused by new mutation in MMACHC. Pediatr. Int..

[21] Sharma A.P., Greenberg C.R., Prasad A.N., Prasad C. (2007). Hemolytic uremic syndrome (HUS) secondary to cobalamin C (cblC) disorder. Pediatr. Nephrol..

[22] Carmel R., Bedros A.A., Mace J.W., Goodman S.I. (1980). Congenital methylmalonic aciduria-homocystinuria with megaloblastic anemia: Observations on response to hydroxocobalamin and on the effect of homocysteine and methionine on the deox-yuridine suppression test. Blood.

[23] Baumgartner E.R., Wick H., Maurer R., Egli N., Steinmann B. (1979). Congenital defect in intracellular cobalamin metabolism resulting in homocystinuria and methylmalonic aciduria. I. Case report and histopathology. Helv. Paediatr. Acta.

[24] Geraghty M.T., Perlman E.J., Martin L.S., Hayflick S.J., Casella J.F., Rosenblatt D.S., Valle D. (1992). Cobalamin C defect associated with hemolytic-uremic syndrome. J. Pediatr..

[25] Chenel C., Wood C., Gourrier E., Zittoun J., Casadevall I., Ogier H. (1993). Syndrome hémolytique et urémique néonatal, acidurie méthylmalonique et homocystinurie par déficit intracellulaire de la vitamine B12. Intérêt du diagnostic étiologique. Arch. Françaises Pédiatrie.

[26] Menni F., Testa S., Guez S., Chiarelli G., Alberti L., Esposito S. (2012). Neonatal atypical hemolytic uremic syndrome due to methylmalonic aciduria and homocystinuria. Pediatr. Nephrol..

[27] Kind T., Levy J., Lee M., Kaicker S., Nicholson J.F., Kane S.A. (2002). Cobalamin C disease presenting as hemolytic-uremic syn-drome in the neonatal period. J. Pediatr. Hematol. Oncol..

[28] Francis P.J., Calver D.M., Barnfield P., Turner C., Dalton R.N., Champion M.P. (2004). An infant with methylmalonic aciduria and homocystinuria (cblC) presenting with retinal haemorrhages and subdural haematoma mimicking non-accidental in-jury. Eur. J. Pediatr..

[29] Lerner-Ellis J.P., Anastasio N., Liu J., Coelho D., Suormala T., Stucki M., Loewy A.D., Gurd S., Grundberg E., Morel C.F. (2009). Spectrum of mutations in MMACHC, allelic expression, and evidence for genotype-phenotype correlations. Hum Mutat..

[30] Lerner-Ellis J.P., Tirone J.C., Pawelek P.D., Doré C., Atkinson J.L., Watkins D., Morel C.F., Fujiwara T.M., Moras E., Hosack A.R. (2006). Identification of the gene responsible for methylmalonic aciduria and homocystinuria, cblC type. Nat. Genet..

[31] Morel C.F., Lerner-Ellis J.P., Rosenblatt D.S. (2006). Combined methylmalonic aciduria and homocystinuria (cblC): Pheno-type-genotype correlations and ethnic-specific observations. Mol. Genet. Metab..

